# The Effect of Obesity on Brain Diffusion Alteration in Patients with Obstructive Sleep Apnea

**DOI:** 10.1155/2014/768415

**Published:** 2014-03-03

**Authors:** Rukiye Kilicarslan, Alpay Alkan, Rasul Sharifov, Muhammed Emin Akkoyunlu, Ayse Aralasmak, Abdulkadir Kocer, Levent Kart

**Affiliations:** ^1^Department of Radiology, School of Medicine, Bezmialem Vakif University, Vatan Street, Aksaray, 34400 Istanbul, Turkey; ^2^Department of Pulmonology, School of Medicine, Bezmialem Vakif University, Vatan Street, Aksaray, 34400 Istanbul, Turkey; ^3^Department of Neurology, School of Medicine, Bezmialem Vakif University, Vatan Street, Aksaray, 34400 Istanbul, Turkey

## Abstract

*Purpose.* We investigated diffusion alterations in specific regions of the brain in morbid obese, obese, and nonobese OSA patients and searched whether there is a correlation between BMI and ADC values. *Materials and Methods.* DWIs of 65 patients with OSA were evaluated. The patients were classified according to BMI as morbid obese (*n* = 16), obese (*n* = 27), and nonobese (control, *n* = 22) groups. ADC measurements were performed from 24 different regions of the brain in each patient. The relationship of BMI with ADC values was searched. *Results.* The ADC values in hypothalamus, insular cortex, parietal cortex, caudate nucleus, frontal white matter, and posterior limb of internal capsule were all increased in obese patients (*n* = 43) compared to control group. The ADC values of midbrain, hypothalamus, orbitofrontal cortex, and parietal cortex were significantly increased in morbid obese compared to obese patients. In obese patients, the degree of BMI was positively correlated with ADC values of orbitofrontal cortex, parietal cortex, and hypothalamus. *Conclusion.* We observed increasing brain vasogenic edema with increasing BMI, suggesting that the main reason of brain diffusion alteration in patients with OSA could be obesity related.

## 1. Introduction 

Obstructive sleep apnea (OSA) is a disease of repeated episodes of upper airway obstruction during sleep, resulting in intermittent hypoxemia and arousal [1]. OSA is related to obesity, diet, or physical inactivity but obesity is a major risk factor. The prevalence is highest among men aged 40–65 years [2, 3]. It usually occurs in individuals who are overweight although the disorder can affect anyone [4]. Patients suffering from OSA are not exclusively obese but 90% of obese patients have sleep apnea [2]. OSA is accepted as a type of disease inducing and exacerbating cognitive deficit and other metabolic disorders if untreated [4].

Neuroimaging studies have showed structural changes in the brains of individuals with OSA [5–11]. Diffusion-weighted Imaging (DWI) enables valuable information in certain pathologies of the brain. Movement of water molecules in the brain is evaluated as qualitatively on DWI and quantitatively on their corresponding apparent diffusion coefficient (ADC) maps. In the literature, there is no study investigating the relationship between obesity and DWI changes in the brain of patients with OSA, to our knowledge.

The purpose of our study is to investigate diffusion alterations in specific regions of the brain among morbid obese, obese, and nonobese patients with OSA and to show whether there is a relation between Body Mass Index (BMI) and ADC values in these patients.

## 2. Materials and Methods

A total of 65 patients (20 females, 45 males, mean age: 48.09 ± 12.35 years) were enrolled into the study. They all were previously referred to our Sleep Clinic Department and diagnosed as OSA based on standard clinical criteria. All subjects underwent a standard all-night polysomnography recording. Apnea index (AI), hypopnea index (HI), and respiratory disturbance index (RDI) were used to assess subjective sleepiness. None of the subjects had cerebrovascular disease, hypertension, dyslipidemia, ischemic changes, leukoaraiosis, and cerebral and cerebellar atrophy. Other exclusion criteria were under medication that could interfere with sleep or cognitive efficiency, neurological disease, hypothyroidism, alcohol or drug abuse, and other sleep disorders that cause similar changes in brain and benign increased intracranial pressure. Neither of the patients has taken continuous positive airway pressure (CPAP) treatment prior to our study.

The BMI was calculated by dividing the weight in kilograms by the square of heighth in meters. BMI groups were defined using the World Healt Organization (WHO) classification system. The patients with BMI more than 30 kg/m² (group 1, *n* = 43, mean age: 50.23 ± 12 years) were accepted as all obese group and categorized into two subgroups according to BMI. Subgroup 1 consisted of 16 morbid obese patients (BMI ≥ 40 kg/m², mean age: 47.25 ± 13 years), and subgroup 2 consisted of 27 obese patients (BMI = 30–39 kg/m², mean age: 52.00 ± 11 years). BMI less than 30 kg/m² was accepted as nonobese and control group (group 2, *n* = 22, mean age: 43.91 ± 11 years). The procedures used were in accordance with the guidelines of the Declaration of Helsinki on human experimentation. The study protocol was approved by the institutional ethical committee. All subjects were fully informed and gave their written informed consent.

**Figure 1:**
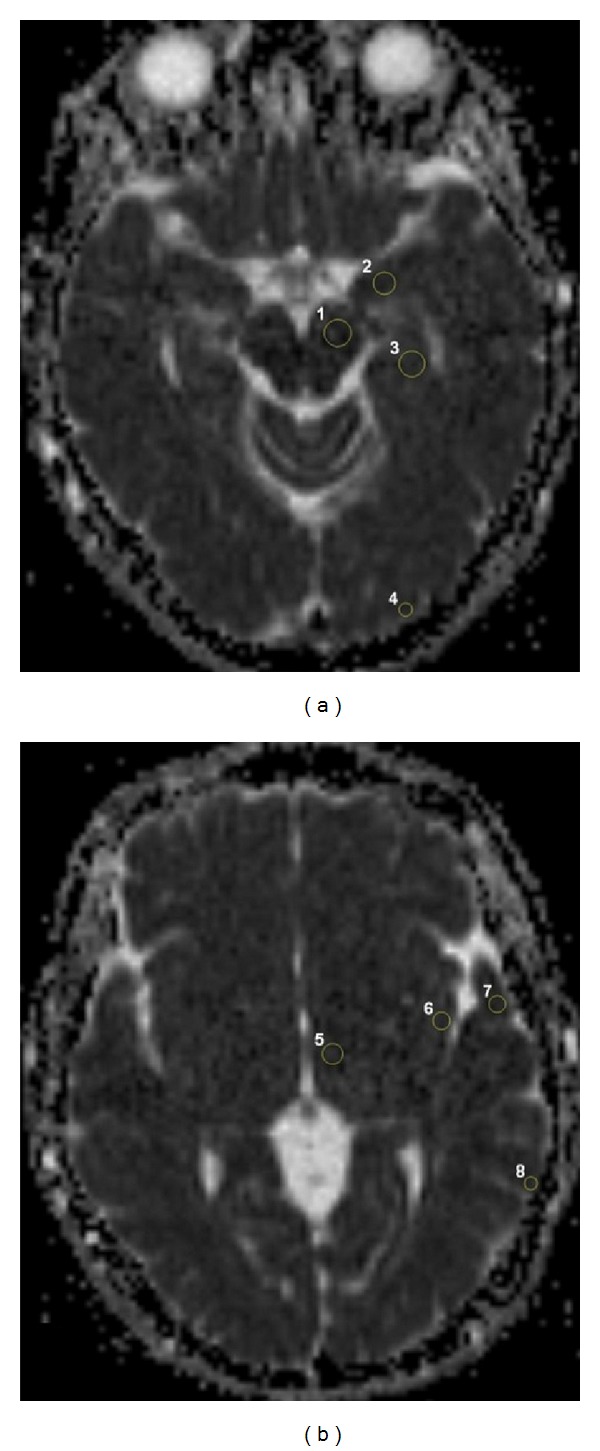
ADC maps show several ROIs: midbrain (1), amygdala (2), hippocampal gyrus (3), and occipital cortex (4) on (a); hypothalamus (5), insular (6), middle temporal (7), and parietal cortices (8) on (b).

**Figure 2:**
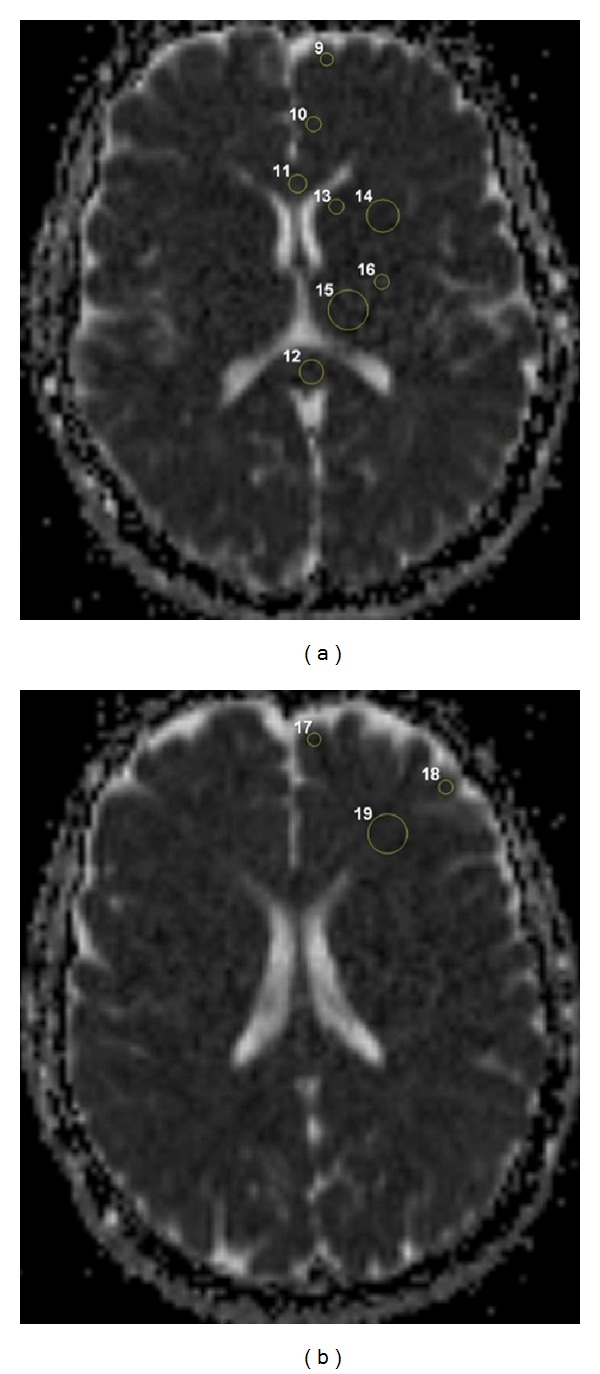
ADC maps show several ROIs: orbitofrontal cortex (9), cingulate gyrus (10), genu (11) and splenium (12) of corpus callosum, caudate nucleus (13), putamen (14), thalamus (15), and posterior limb of internal capsula (16) on (a); dorsomedial (17) and dorsolateral (18) frontal cortices and frontal white matter (19) on (b).

The Magnetic Resonance Imaging (MRI) examination consisted of routine imaging including DWI. MRI was performed on 1.5-T system (Siemens, Avanto, Erlangen, Germany). Fast spin echo T2WI (TR = 4530 ms, TE = 100 ms) in the axial and sagittal planes, T1WI (TR = 550 ms, TE = 20 ms) in the axial plane, and FLAIR images (TR = 8000 ms, TE = 90 ms) in the axial and coronal planes were obtained. For DWI, a single-shot spin echo-planar pulse sequence (TR = 6900 ms, TE = 89 ms, field of view (FOV) = 230 mm, matrix size = 128 × 128, number of acquisitions = 2, slice thickness = 5 mm, slice number = 25, slice orientation = axial plane, scan time = 8 s, and interslice gap = 1 mm) was used in all patients with two different *b*-values (0 and 1000 s/mm^2^). The ADC maps were reconstructed with the commercially available software. In all groups, 24 distinct neuroanatomic locations were selected for the analysis. These anatomic locations were previously suggested to be related to satiety-hunger and cognitive function [12–16]. Circular region of interests (ROI) was drawn manually by an experienced radiologist from the previously identified regions. Their ADC values were calculated from the corresponding ADC map by automatical and voxel-wise approach. The sizes of ROIs were 110–130 mm^2^ in thalamus and putamen, 60–80 mm^2^ in dentate nuclei, midbrain, amygdala, hippocampal gyrus, hypothalamus, globus pallidus, and frontal white matter, 30–50 mm^2^ in vermis, genu and splenium of corpus callosum, optic radiation, and cingulate gyrus, and 10–30 mm^2^ in cortices (cerebellar, occipital, parietal, middle temporal, insular, orbitofrontal, dorsomedial frontal, and dorsolateral frontal), caudate nucleus and posterior limb of internal capsula. We minimized partial volume effects and avoided averaging with cerebrospinal fluid (CSF) by inspecting the slices above and below the region and using smaller ROI in cortical areas. ROIs maps in different brain regions were presented in Figures 1 and 2. The radiologists carefully evaluated the selected regions in all patients by similar ROI's size. The mean ADC values of measurements performed by two radiologists from the same areas were taken under consideration. ROI analyzers were blinded to the condition of the subjects.

All statistical analyses were performed using a commercially available SPSS release 20.0 software package (SPSS Inc., Chicago, IL, USA). The results were presented as the mean ± standard deviation (SD). To test differences between groups, Kruskal-Wallis variance analysis was used. The groups showed normal distribution. In evaluation of sociodemographic variables, ANOVA (Tukey's test) was used. Student *t*-test was used for the comparison of the groups. Pearson test was used for correlation analysis. A *P* value below 0.05 was considered statistically significant.

## 3. Results

Demographic information and clinical information were presented in Table 1 and mean ADC values of each group in 24 different brain regions were presented in Table 2.

There was no significant difference between age, gender, RDI, and AI values of subgroup 1, subgroup 2, all obese group, and control group. There was a significant difference between HI values of morbid obese and control group (*P* = 0.001) and between HI values of obese and control group (*P* = 0.006).

**Figure 3:**
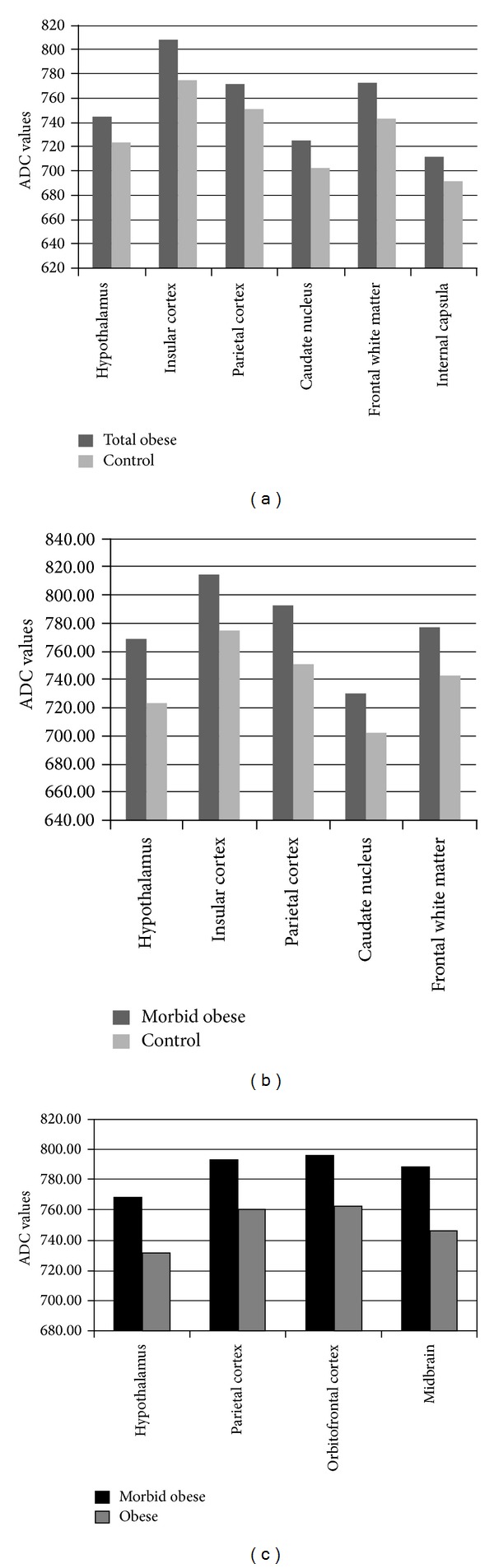
(a) The figure shows that the ADC values (×10^−6 ^mm²/s) in hypothalamus, insular cortex, parietal cortex, caudate nucleus, posterior limb of internal capsula, and frontal white matter were significantly increased in total obese patients compared to control group. (b) The figure shows that the ADC values (×10^−6 ^mm²/s) in hypothalamus, insular cortex, parietal cortex, caudate nucleus, and frontal white matter were significantly higher in morbid obese compared to control group. (c) The figure shows that the ADC values (×10^−6 ^mm²/s) in midbrain, hypothalamus, orbitofrontal cortex, and parietal cortex were significantly increased in morbid obese compared to obese patients.

The ADC values in hypothalamus (*P* = 0.004), insular cortex (*P* = 0.004), parietal cortex (*P* = 0.000), caudate nucleus (*P* = 0.005), posterior limb of internal capsula (*P* = 0.003), and frontal white matter (*P* = 0.003) were increased in all obese patients compared to control group (Figure 3(a)).

The ADC values in hypothalamus (*P* = 0.000), insular cortex (*P* = 0.008), parietal cortex (*P* = 0.000), caudate nucleus (*P* = 0.030), and frontal white matter (*P* = 0.008) were significantly higher in morbid obese compared to control group (Figure 3(b)).

The ADC values of midbrain (*P* = 0.005), hypothalamus (*P* = 0.002), orbitofrontal cortex (*P* = 0.029), and parietal cortex (*P* = 0.003) were significantly increased in morbid obese (subgroup 1) compared to obese (subgroup 2) patients (Figure 3(c)).

The ADC values of frontal white matter (*P* = 0.017) only was significantly increased in obese patients compared to control group.

In all obese patients with OSA (*n* = 43), the degree of BMI was positively correlated with ADC values of hypothalamus (*r* = 0.51, *P* = 0.000), orbitofrontal cortex (*r* = 0.49, *P* = 0.001), and parietal cortex (*r* = 0.36, *P* = 0.015). There is a positive correlation between RDI and ADC values in occipital cortex (*r* = 0.43, *P* = 0.003), middle temporal cortex (*r* = 0.31, *P* = 0.035), and insular cortex (*r* = 0.32, *P* = 0.032) in all obese patients with OSA. In all obese patients with OSA (*n* = 43), the degree of AHI was positively correlated with ADC values of occipital cortex (*r* = 0.42, *P* = 0.005) and insular cortex (*r* = 0.32, *P* = 0.035).

There is no effect of age on ADC values in all groups including subgroups.

## 4. Discussion

OSA is the most common type of apnea, and it develops gradually overtime. It is related to intermittent obstructions of upper airway throughout sleep. The prevalence of OSA in adult is 2–4% [1]. This ratio is similar worldwide and does not show regional differences [12]. OSA begins as benign snoring and increasing with weight and age [13].

Excessive daytime sleepiness is one of the most frequent symptoms identified in patients with OSA. The residual sleepiness could be a posthypoxic injury phenomenon or a specific neuropsychological syndrome [3]. Repetitive episodes result in decreased arterial oxygen saturation and transient arousals with marked disruption of normal sleep architecture as much as 40% or more in severe cases. There is a correlation between changes in brain and clinical findings in OSA. The brain responds to the lack of oxygen by alerting the body, causing a brief arousal from sleep that restores normal breathing [13]. Some studies [5, 14] have shown an association between the presence of OSA and an increased risk of developing mild cognitive impairment or dementia, whereas Quan et al. found that there is no association between the severity of OSA and the neurocognitive function. They thought that OSA causes cognitive changes in only subsets of individuals with OSA [6].

Obesity is the most important risk factor for OSA. The majority of adult patients with OSA are obese. The percentage of children diagnosed with OSA is increasing as obesity rate widened [4]. Obesity compromises the upper airway size by fatty infiltration along the pharyngeal walls and within the tongue and limiting chest dimensions and abdominal movements. Because of the relationship between obesity and OSA, neuroimaging investigations were performed for satiety- and hunger-related centers and cognitive centers [12]. Although obesity is often reported in previous studies related to OSA, the effect of obesity on observed findings is generally not explored nor discussed [17]. RDI, HI, and AI are used to diagnose and characterize the severity of OSA. In our study, RDI and AI values were not significantly increased in all obese patients compared to nonobese patients with OSA. HI values were more prominent in morbid obese. These findings suggest that there is a relationship between severity of OSA and obesity.

Until now, many patients with OSA were unable to appropriately fit into the MRI scanner. This limits interpretation regarding the interaction of OSA and obesity and/or any pathophysiological differences of OSA among obese and nonobese patients [17]. Currently, with developing MRI technologies, especially morbid obese patients are easily tolerating MRI scanning and in our study, all patients had undergone MRI scanning successfully.

Advances in neuroimaging technology have given us the ability to evaluate the brain functions. DWI is an important tool in the neurosciences to understand the key structure-function relationship of the brain. In DWI, image contrast occurs via the molecular motion of water, which may be substantially altered in diseases [15]. DWI reliably distinguishes vasogenic edema from cytotoxic edema. Hypoxia-related decrease in the value of ADC is a result of cell swelling and reductions in extracellular space. ADC increase is associated with reduced cell volumes and increased extracellular space. Increased ADC values suggest ultrastructural changes and, therefore, would reflect microstructural damage [18]. Few studies so far have used DWI in obesity [19, 20]. Alkan et al. reported that ADC values of distinct brain locations related to satiety and hunger were significantly increased in obese individuals. The ADC changes in the same locations were more prominent in morbid obese [19]. Moreover, several studies revealed significant correlation between BMI and diffusion changes [19, 20].

Neuronal damage and vasogenic edema in the different brain regions of OSA patients due to intermittent hypoxia have been reported in the literature [7, 8, 17, 21]. In recent studies about OSA, gray matter loss has been reported in different brain regions including the cortical gray matter, hippocampus, frontal and parietal cortex, temporal lobe and anterior cingulated cortex with cerebellum affected in severe diseases. Their findings suggested that significant cognitive impairment in patients with OSA is associated with brain tissue damage [9, 22–25].

In our study, the ADC values of cognitive centers were significantly increased in morbid obese when compared to nonobese control patients with OSA. These findings pointed out that morbid obesity is associated with altered extracellular fluid (ECF)/intracellular fluid (ICF) ratio in cognitive centers. Moreover, the ADC values of midbrain, hypothalamus, orbitofrontal cortex, and parietal cortex were significantly increased in morbid obese when compared to obese patients with OSA. Moreover, demonstration of the positive correlation between BMI and ADC values in hypothalamus, orbitofrontal cortex, and parietal cortex supports the importance of diffusion alteration in obesity with OSA. Overall, our findings suggest that there is a parallel increase between BMI and altered fluid ECF/ICF ratio in different brain locations in overweighted patients with OSA. This may be due to vasogenic edema, gliosis, cell death, or metabolic changes. The mechanism is not yet clear in this study.

Excessive daytime sleepiness is the main cause of the neuropsychological deficits in patients with OSA and the comorbidities, such as cardiovascular disease, obesity, and physical inactivity usually are more important than the sleep apnea [23]. The presence of OSA seems to be a factor accelerating the brain aging process [9]. However, Torelli et al. [9] hypothesized that brain damage in OSA is independent of age, gender, use of tobacco, and cardiovascular comorbidities. In our patients with OSA, compatible with Torelli's result, we could not find any effect of age on ADC values and we noticed a strong correlation between increasing ADC values and the severity of obesity in many different regions of the brain.

The recent study by Kumar et al. have shown a decrease in global and regional mean diffusivity in the brain in OSA [26]. They attributed this finding to axonal, glial, and other cell changes. They also found that certain areas were more affected. Consistent with these findings, in our study, the increase in ADC values reflects microstructural damage in specific brain areas.

## 5. Conclusion

We found microstructural changes in the brain in OSA patients which could be related to obesity and/or hypoxia, and increasing ADC values with increasing BMI. We hypothesize that the main reason of brain diffusion changes in patients with OSA could be obesity related. Although the mechanism is not yet clear, brain diffusion alteration in patients with OSA patients may help in the understanding of the underlying pathophysiology and guide new treatment strategies.

## Figures and Tables

**Table 1:** Demographic and clinical characteristics in patients with OSA.

Patients	Age	RDI	AI	HI
Group 1 (*n* = 43)	50.23 ± 12	38.93 ± 26	29.65 ± 26	54.47 ± 38
Subgroup 1 (*n* = 16)	47.25 ± 13	38.75 ± 28	27.44 ± 27	62.69 ± 43
Subgroup 2 (*n* = 27)	52.00 ± 11	39.04 ± 26	30.96 ± 26	49.59 ± 35
Group 2 (*n* = 22)	43.91 ± 11	26.41 ± 21	23.09 ± 20	18.73 ± 21

Group 1: total obese patients (BMI ≥ 30 kg/m²).

Subgroup 1: morbid obese patients.

Subgroup 2: obese patients.

Group 2: nonobese (control) patients.

RDI: respiratory disturbance index.

AI: apnea index.

HI: hypopnea Index.

**Table 2:** Mean ADC values (×10^−6^ mm²/s) of different brain regions in patients with OSA.

Locations	Group 1 (*n* = 43)			Group 2 (*n* = 22)
	Subgroup 1 (*n* = 16)	Subgroup 2 (*n* = 27)	Total (*n* = 43)	
Dentate nucleus	696.60 ± 29	713.04 ± 25	706.93 ± 27	704.05 ± 13
Cerebellar vermis	724.06 ± 31	744.48 ± 46	736.88 ± 42	721.00 ± 25
Cerebellar cortex	741.00 ± 34	742.22 ± 37	741.77 ± 35	748.73 ± 38
Amygdala	787.75 ± 38	796.19 ± 34	793.05 ± 36	795.23 ± 41
Midbrain	788.50 ± 32	746.22 ± 45	761.95 ± 45	764.36 ± 40
Hippocampal gyrus	831.19 ± 33	828.30 ± 40	829.37 ± 37	812.91 ± 35
Occipital cortex	789.19 ± 26	784.04 ± 26	785.95 ± 26	775.32 ± 31
Hypothalamus	768.69 ± 33^a,b^	731.11 ± 31	745.09 ± 36^d^	723.45 ± 34
Middle temporal cortex	763.56 ± 36	759.19 ± 44	760.81 ± 41	750.00 ± 51
Insular cortex	814.50 ± 36^b^	805.11 ± 29	808.60 ± 32^d^	774.82 ± 48
Parietal cortex	792.75 ± 29^a,b^	760.11 ± 31	772.26 ± 34^d^	750.77 ± 28
Orbitofrontal cortex	795.38 ± 40^a^	761.85 ± 38	774.33 ± 41	771.32 ± 40
Cingulate gyrus	794.56 ± 38	788.78 ± 36	790.93 ± 36	785.18 ± 26
Posterior limb of internal capsula	714.56 ± 43	710.89 ± 33	712.26 ± 37^d^	691.95 ± 22
Caudate nucleus	730.25 ± 37^b^	722.07 ± 22	725.12 ± 28^d^	702.64 ± 36
Globus pallidum	745.31 ± 47	737.85 ± 40	740.63 ± 42	730.68 ± 31
Putamen	745.25 ± 27	741.41 ± 36	742.84 ± 32	734.59 ± 26
Thalamus	751.13 ± 46	754.67 ± 31	753.35 ± 36	750.59 ± 30
Optic radiation	781.88 ± 44	780.89 ± 33	781.26 ± 37	769.64 ± 29
Genu of corpus callosum	772.44 ± 46	778.44 ± 38	776.21 ± 41	778.59 ± 42
Splenium of corpus callosum	760.94 ± 58	759.81 ± 51	760.23 ± 53	751.68 ± 47
Dorsomedial frontal cortex	753.38 ± 36	774.11 ± 51	766.40 ± 47	778.73 ± 55
Dorsolateral frontal cortex	755.81 ± 41	769.30 ± 34	764.28 ± 37	796.68 ± 60
Frontal white matter	777.31 ± 38^b,c^	770.63 ± 30	773.12 ± 33^d^	743.27 ± 32

Group 1: total obese patients (BMI ≥ 30 kg/m²).

Subgroup 1: morbid obese patients.

Subgroup 2: obese patients.

Group 2: nonobese (control) patients.

^a^Significant differences between subgroup 1 and subgroup 2.

^b^Significant differences between subgroup 1 and group 2.

^c^Significant differences between subgroup 2 and group 2.

^d^Significant differences between group 1 and group 2.
